# Sonopharmacology: controlling pharmacotherapy and diagnosis by ultrasound-induced polymer mechanochemistry

**DOI:** 10.1039/d2sc05196f

**Published:** 2022-11-07

**Authors:** Deniz Yildiz, Robert Göstl, Andreas Herrmann

**Affiliations:** DWI–Leibniz Institute for Interactive Materials Forckenbeckstr. 50 52056 Aachen Germany herrmann@dwi.rwth-aachen.de goestl@dwi.rwth-aachen.de; Institute of Technical and Macromolecular Chemistry, RWTH Aachen University Worringerweg 1 52074 Aachen Germany

## Abstract

Active pharmaceutical ingredients are the most consequential and widely employed treatment in medicine although they suffer from many systematic limitations, particularly off-target activity and toxicity. To mitigate these effects, stimuli-responsive controlled delivery and release strategies for drugs are being developed. Fueled by the field of polymer mechanochemistry, recently new molecular technologies enabled the emergence of force as an unprecedented stimulus for this purpose by using ultrasound. In this research area, termed sonopharmacology, mechanophores bearing drug molecules are incorporated within biocompatible macromolecular scaffolds as preprogrammed, latent moieties. This review presents the novelties in controlling drug activation, monitoring, and release by ultrasound, while discussing the limitations and challenges for future developments.

## Abstract 2

Ultrasound is an important tool for diagnostic and therapeutic purposes in the clinic. We here survey recent efforts to exploit the principles of polymer mechanochemistry for ultrasound-guided drug delivery and activation – sonopharmacology.

## Graphical abstract

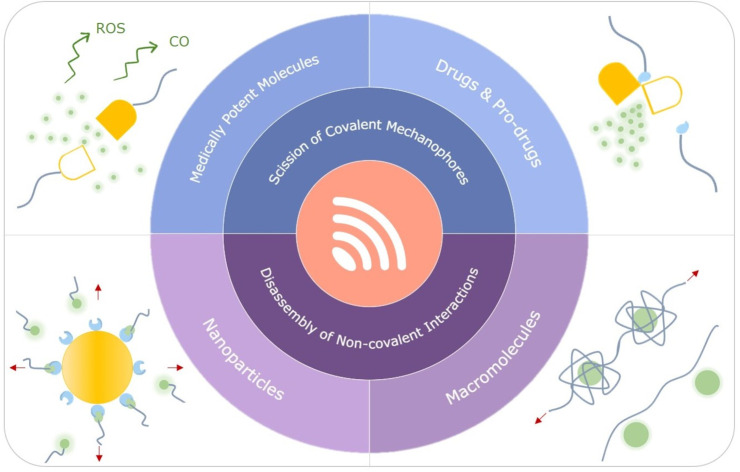

## Introduction

Medical professionals use ultrasound (US) as an important tool for diagnostic and therapeutic purposes. In the last decades, US has gained significant attention as a controlling appliance to overcome systematic limitations of pharmacotherapy, that are drug resistance,^1,2^ environmental toxicity,^3^ degradation,^4^ and most importantly off-target activity.^5,6^ In contrast to other exogenous stimuli,^7,8^ such as temperature,^9^ light,^10,11^ and magnetic fields,^12^ US is attractive because of its benign nature and its interactions with biological media, inducing the uptake of impermeable molecules or causing destruction to release payloads.^13^

US is defined as periodic vibration sound waves with a frequency above 20 kHz.^14^ These sound waves can be used as a unique diagnostic modality providing non-invasive real-time imaging in medicine.^15^ Alongside, US has been used therapeutically since the beginning of the 20th century, offering many advantages, such as ease of application and spatiotemporal control.^16–18^ Medical US is classified into three categories based on frequency and applications: low frequency US describes sound waves below 1 MHz, which can be used for sonophoresis, transdermal permeability enhancements, sonobactericide, and tissue ablation.^19,20^ Application safety increases with frequency because of the diminished damage to and overheating of tissues. Medium frequency US lies between 1 and 5 MHz and high frequency US describes sound waves above 5 MHz. Both are mainly used for either theranostic or purely diagnostic purposes.^21^ Focused US with either high (HIFU) or low (LIFU) power intensity has been recently developed to allow theranostic applications and enhance the usage of tissue ablation as well as targeted drug delivery.^22^ US interacts with the biological media twofold, thermally and non-thermally. Thermal interactions are acoustic heating and streaming, caused by acoustic energy transfer to increase temperature in the media. Nonthermal interactions, on the other hand, are based on cavitation.^23^ Cavitation originates from the oscillation of air bubbles under periodic acoustic pressure and the resulting collapse of the bubbles exerts shear force in the surrounding media. This shear force can disrupt the drug-releasing carriers and cause synergistic effects, such as enhancing drug uptake and permeabilization.

Major efforts on the utilization of US for biomedical applications are focused on imaging and release involving loaded cargo moieties, such as liposomes,^24,25^ nanobubbles,^26–28^ micelles,^29,30^ and microbubble based agents.^21,31,32^ These carriers are therefore used for therapeutic action promoted by US-induced intracellular transportation of molecular drugs, based on the enhanced penetration and retention (EPR) effect and enhanced permeabilization by sonoporation. Microbubbles have been used and advanced for imaging modalities, as they are promising contrast agents for detection and characterization within biological environments.^33^ Over and above, microbubbles can induce oscillations and cavitation in the media under US, subsequently leading to acoustic forces that are currently used to increase drug permeability and efficacy in medical research. Different internalization routes, resulting from different ultrasound settings and correlative microbubble behavior, allow tuning pore sizes to corresponding cargo sizes.^34^ Low intensity ultrasound causes stable cavitation of microbubbles, forming microstreams around the microbubbles that allow the uptake of small molecules by formation of small pores and endocytosis on the cells in close vicinity. High intensity ultrasound, on the other hand, induces collapse of microbubbles due to inertial cavitation and causes shock waves and microjets which perforate cell membranes allowing direct cytoplasmic entry of larger drugs. As these modalities are applied to more realistic tumor models, it has been shown that ultrasound, with or without microbubbles, affects not only the cell membranes, but also the tumor microenvironment, *i.e.*, extracellular matrix and vasculature, thermally and mechanically, altering interstitial fluid pressure, which is a major barrier to drug delivery in solid tumors.^35^ Not only microbubbles, but also liposomes and micelles are of great interest in targeted therapies as they can carry high concentrations of drugs and shield them at the same time. Sonoporation-assisted therapeutic action and microstreaming-amplified disassembly have been eminently reported in the last decades,^36,37^ however, specific and selective chemical transformations in biological environments or tissues induced by US remained elusive.

In the field of polymer mechanochemistry, such chemical reactions have already been realized. Cavitation-originated shear force in solution can lead to conformational, configurational, and constitutional rearrangements in macromolecular systems.^38,39^ To activate such force-induced events, mechanically labile molecular motifs (mechanophores) must be tailored meticulously and anchored within macromolecular frameworks. A variety of different applications has been developed with such moieties being employed for optical sensing of force-induced events,^40,41^ mechanically triggered catalysis,^42^ and molecular release.^43,44^ Specifically the latter has become an active research area over the last years, since US is, in principle, a straightforward stimulus for biological media and tissues to selectively address the functional motifs, enabling control over spatial and temporal dosage of drugs.

These exclusive advantages render the application of the principles of polymer mechanochemistry for pharmacotherapy (sonopharmacology) a promising technology compared to existing techniques. Photopharmacology,^10,11^ for instance, has the advantage of the unparalleled spatiotemporal resolution of light and is principally non-invasive. However the maximum depth of light penetration is quite limited to the millimeter range within the phototherapeutic window.^45^ This occasionally requires light-based therapies to rely on minimal invasive surgeries to bring the light source to the target area. Ultrasound, on the other hand, can penetrate biological tissues by tuning frequency and power intensity up to multiple centimeters.^46^ Additionally, photopharmacology relies on photoswitches as responsive units, which may lack biocompatibility and solubility in biological media. Conversely, sonopharmacological agents can be prepared from purely biological macromolecules, such as nucleic acids, or bio-compatible, non-cytotoxic macromolecules.

However, certain pitfalls must be overcome to eventually apply sonopharmacology. For example, regular polymer mechanochemistry generally relies on low frequency (around 20 kHz) US that produces inertial cavitation. While some medical applications, *e.g.*, tissue ablation and thrombolysis, make use of such US conditions,^20^ they can hardly be regarded as benign. Yet, the utilization of HIFU^22^ or microbubbles^32^ was shown to reduce sonication damage and overheating in tissues by lowering the cumulative energy input. In addition, apparently contradictory properties, such as the loaded cargo content and the mechanochemical activity expressed through the contour length and thus molar mass of the carrier polymer, must be balanced. It hence becomes obvious that this field is in rapid motion and continuously develops rendering an account over the current state-of-the-art highly desirable.

**Fig. 1:**
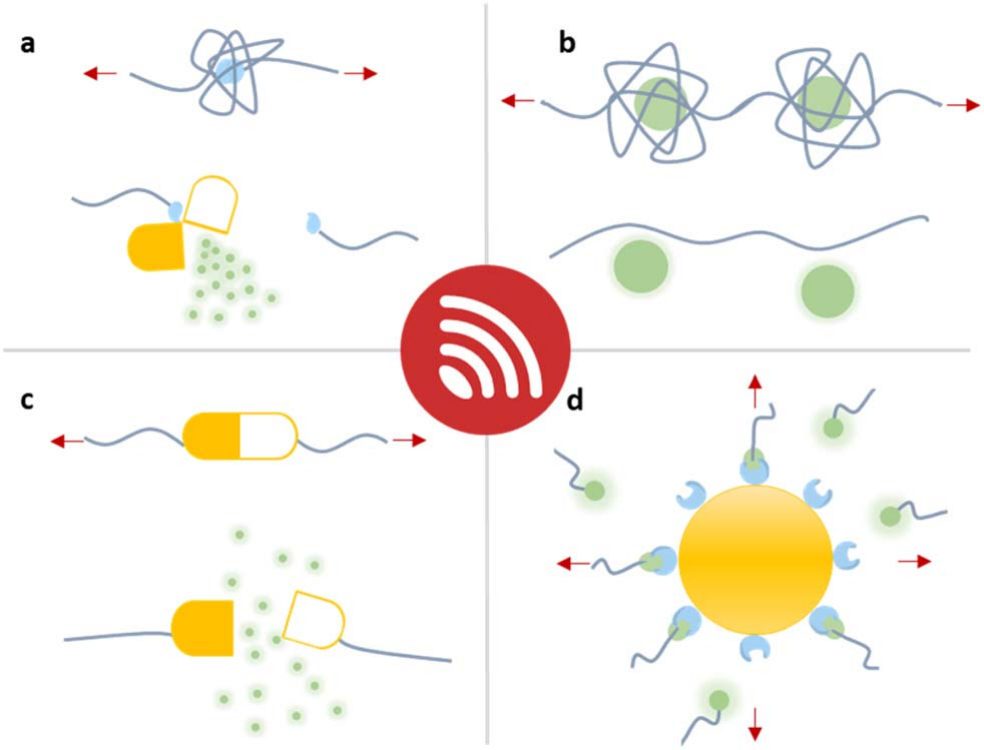
Schematic depiction of the strategies that have been developed for the mechanochemical release of drug molecules from macromolecules. Entrapped payloads can be released through (a) covalent bond scission, (b and c) non-covalent macromolecular disassembly, and (d) nanoparticle disaggregation, all requiring different US doses.

In this overview article, we will present the recent achievements that have fostered the new field “sonopharmacology” and discuss its potentials and limitations. We will first discuss developments in sonication-induced cargo release based on the scission of covalent mechanophores (Fig. 1a). Then, we will describe novel host–guest mechanophore systems that consist of macromolecules (Fig. 1b and c) or nanoparticles (NPs) (Fig. 1d) assembled by non-covalent interactions. The overall aim and scope of this review is to engage readers with a strong synthetic chemical, polymer chemical, biochemical, and pharmaceutical research background by providing an overview of US-induced drug activation on the molecular level and related release methods.

## The mechanochemical activation of macromolecules

Polymer mechanochemistry using mechanophores allows activation of molecular function with high spatiotemporal precision and can be readily transferred to many macromolecular systems. Mechanophore activation is based on a weakening strategy by employing labile motifs in comparatively stronger polymeric structures. When force is applied on polymeric materials in the form of compression, extension, swelling, freezing, contraction flow, or shearing in solution,^47^ this induces molecular transformations, such as conformational changes or bond scission, eventually activating latent functionality.^38,39^

To induce molecular events by force, the mechanophores must be tailored to and anchored within the respective polymeric architecture. In bulk systems, mechanophores can be implemented as crosslinkers while in linear polymers, the mechanophore should be placed in the vicinity of the chain center so that the pulling forces cause full or partial elongation of the polymeric backbone followed by mid-chain scission, where the force accumulation is the highest.^48^ Beyond the location of the mechanophore, mechanochemical transformations highly depend on the molar mass of the linear chain or chain segment and chains are inert to force below a limiting molar mass *M*_lim_ better expressed as degree of polymerization *X*_lim_ or contour length *L*_lim_.^49,50^ This means that longer polymer chains show higher mechanochemical activity while at the same time they lead to a decreasing loaded drug content. This may be circumvented by using polymer architectures with pronounced and high mechanochemical activity, such as brushes,^51–53^ microgels,^54–57^ or dendritic systems.^58–60^ Alternatively, non-scissile multi-mechanophore architectures may be employed to avoid the mechanochemical deactivation of the carrier after the first release event.^61,62^

The implementation of covalent or non-covalent mechanophores in macromolecular structures facilitates targeted therapy by means of quantity, duration, and location of treatment. In most cases, this entails the force-induced release^43,44^ of drug molecules from their macromolecular carriers, but occasionally chain-terminal functionalities or NPs are used. Although the systems presented in the following may not fulfill all necessary conditions for their translation into medical applications yet, they mark crucial milestones and point towards possible future developments in the field of sonopharmacology.

## Covalent mechanophore systems

### Releasing or activating gases for medical applications

**Fig. 2:**
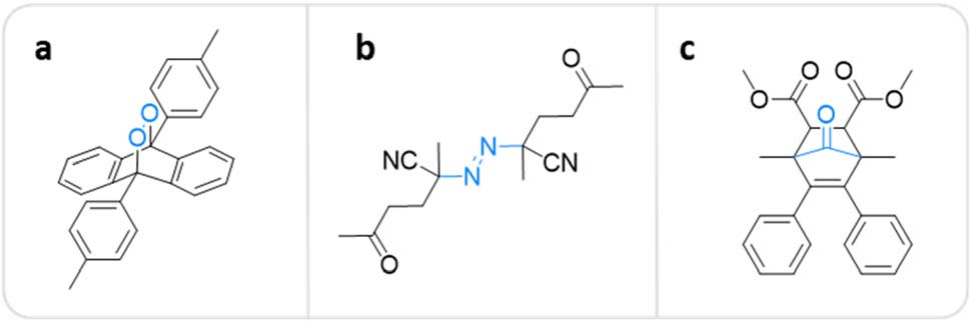
Mechanophore structures releasing or generating diatomic molecules under mechanical force. (a) 9,10-Diphenylanthracene-endoperoxide releasing ^1^O_2_.^71^ (b) Azo-based mechanophore generating FRs and ROS.^74^ (c) Norborn-2-en-7-one releasing CO.^78^

A polymer equipped with mechanophores can break into smaller oligomers that can function as active sites for catalysis,^63–65^ optical sensing,^66–68^ and many other applications.^69,70^ For pharmacotherapy, however, the release of small bioactive molecules is a crucial feature.^10,11,43,44^ The first mechanochemical system potentially useful in this context was developed by Baytekin, Akkaya, and coworkers.^71^ Singlet oxygen (^1^O_2_), which belongs to the family of reactive oxygen species (ROS) with a μs lifetime,^72,73^ was generated from poly(methyl acrylate) (PMA) and poly(dimethylsiloxane) (PDMS) scaffolds *via* cycloelimination from a 9,10-diphenylanthracene-endoperoxide crosslinker under shear force (Fig. 2a). However, this system was not applicable in a medical context, because the form of applied mechanical force, *i.e.*, compression and cryogenic ball milling, was not feasible in biological environments, and also may cause elevation of temperature on the applied surfaces.

Overcoming this limitation, Li, Moore, and coworkers recently incorporated azo-based mechanophores as crosslinkers into biocompatible PEG hydrogel networks that can be activated by a clinically validated modality, namely HIFU.^74^ Irradiation of the hydrogels with HIFU (550 kHz) resulted in covalent chain scission resulting in free radical generation, and the subsequent formation of ROS (Fig. 2b). Luminol chemiluminescence and xylenol orange/Fe^2+^ colorimetric tests confirmed the ROS production. To further validate the therapeutic availability of this system, *in vitro* studies were performed on two widely used cancer cell lines – melanoma (B16–F10) and breast cancer (EO771). Hydrogels that are stable under physiological conditions showed inhibition of growth and decrease in viabilities upon activation with HIFU irradiation as short as 40 s. 72 h of incubation resulted in death rates close to 100%, while untreated control EO771 cells proliferated over 400%. The proposed methodology allows using azo mechanophores as a free radical (FR) source to generate ROS in the context of sonodynamic therapy comparable with lethal doses of H_2_O_2_ with a milder irradiation setup that can be tuned to meet biomedical requirements.

Beyond ROS, carbon monoxide (CO) is another potential prophylactic and therapeutic agent when administered in low concentrations, in contrast to its toxicity in high concentrations.^75–77^ Current administration methods of CO in clinical research largely rely on inhalation, which lacks safety and proper dosing. Recently, Moore and coworkers developed a non-scissile CO-releasing norborn-2-en-7-one (NEO) mechanophore that was incorporated in polymers by ring-opening metathesis polymerization (ROMP).^78^ CO can be released either by pulsed US in solution or mortar and pestle in the solid state (Fig. 2c). The authors hypothesized that mechanical force exerted on the polymeric backbone should, in principle, result in selective cleavage of the C_5_–C_6_ bond without backbone scission followed by β-elimination, releasing multiple molecules CO. The mechanochemical release was demonstrated by sonicating the linear polymer with an *M*_n_ of 158.8 kDa for 240 min while monitoring with NMR and fluorescence spectroscopy. The results indicated the emergence of new aromatic peaks on the polymer backbone due to CO release. Above, the CO release was detected qualitatively by gas chromatography, infrared spectroscopy, and thermal gravimetric analysis. The authors determined that up to 154 CO molecules were released per chain and the released amount was directly proportional to the *M*_n_ of the polymer.

The model developments described in this section enable two important features for possible future developments in the clinic: firstly, the controlled release of gases reduces off-target activity. Secondly, the release of multiple units per macromolecule provides a higher loaded cargo content and thus reduces the necessary relative carrier concentration.

### Releasing drugs and prodrugs

**Fig. 3:**
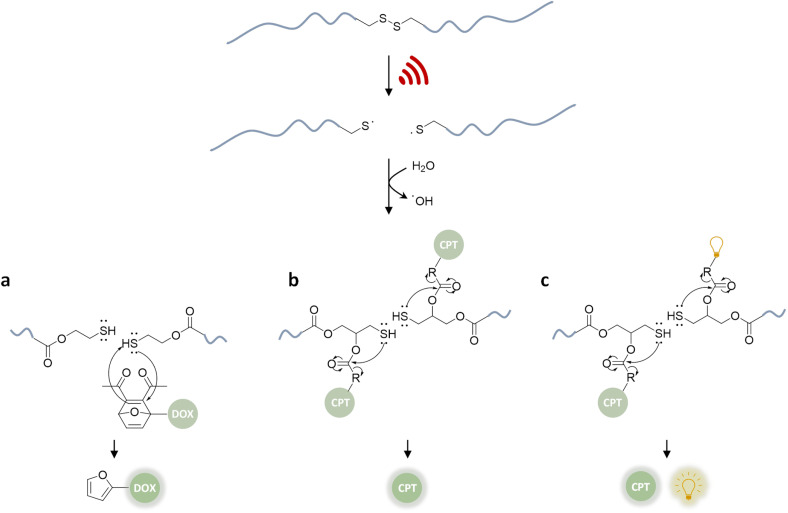
US-induced scission of disulfide-centered polymers and release of drug or reporting molecules. Generated thiols undergo (a) intermolecular Michael-addition to initiate retro Diels–Alder reaction releasing furylated doxorubicin,^79^ (b) intramolecular 5-*exo-trig* cyclization to release camptothecin,^61^ (c) intramolecular 5-*exo-trig* cyclization to release camptothecin and fluorescent umbelliferone simultaneously.^85^

In a pioneering report by Herrmann, Göstl, and coworkers, a release system for furan-containing drugs was described based on the US-induced (20 kHz) homolytic cleavage of disulfide mechanophores embedded centrally within linear polymer chains.^79^ Disulfides have long been established as mechanophores^80–83^ with relatively low bond dissociation energies of *ca.* 270 kJ mol^−1^ compared to C–C bonds with *ca.* 350 kJ mol^−1^.^84^ A disulfide-centered water soluble poly(oligo(ethylene glycol)methyl ether acrylate) (POEGMEA) was synthesized by Cu-mediated controlled radical polymerization to yield a polymer with an *M*_n_ of 48.9 kDa. Initially, US was used to break the polymer chain at the central disulfide site yielding thiyl radicals and then thiols by abstraction of hydrogen radicals from H_2_O (Fig. 3a). Those thiols then underwent Michael addition with the prodrug Diels–Alder adducts, resulting in a retro Diels–Alder reaction to release the furylated drug derivatives. This was first demonstrated with a fluorescent probe, furan–dansyl, for quantification purposes. The sonication experiments were performed *ex situ* and *in situ* to identify and avoid the nonspecific effects of US. The *ex situ* approach was based on sonicating the polymer alone and subsequently mixing it with the prodrug in the presence of a proton scavenger, whereas the *in situ* method was based on mixing all reagents before the sonication. Both pathways illustrated the successful release of up to 70% cargo in 1–3 h sonication time, followed by 72 h of incubation time for completion of the retro Diels–Alder reaction.

The mechanochemical release mechanism was tested with two pharmacologically relevant small molecules, furosemide and doxorubicin. The former is used as a loop diuretic, especially for pediatric patients diagnosed with cardiovascular, pulmonary, and kidney diseases, and the latter is very widely used as a chemotherapy reagent for cancers, such as leukemia, malignant lymphomas, and solid tumors. Furosemide bears the furan moiety as a constituent part of the pharmacophore, while doxorubicin was functionalized with a furan moiety through a carbamate linkage, which is lost at tumor-like pH thus rendering it a prodrug. Both underwent retro Diels–Alder under US. In addition, *in vitro* studies with the doxorubicin derivative demonstrated that the sonicated drug mixture was less effective than pure doxorubicin in killing HeLa cells, yet more effective than the inactive prodrug. Especially for chemotherapeutics, like doxorubicin, controlled release mechanisms are essential for efficient treatment due to their high toxicity and short half-life. However, the substitution of the furan moiety on the drug moiety decreases the potency of the drugs. Combined with the fact that one polymer chain can release two drug molecules, the treatment requires high polymer concentrations. Despite being limited to drugs with a furan moiety, this proof-of-concept work pioneered the sonochemical release of drug molecules.

Besides its narrow applicability, this approach lacked essential properties, such as a unified design in one molecule and real-time tracking of activity. Herrmann, Göstl, and coworkers thus presented a related improved disulfide-centered linear polymer where the drugs were covalently attached to the β-position of the disulfide moiety through a carbonate linker (Fig. 3b).^61^ Upon US irradiation, the linear POEGMEA cleaved to free thiols that subsequently underwent a 5-*exo-trig* cyclization to release the drug molecule from the carbonate linker. The activation was first verified by quantification of the “turn on” fluorescence and absorbance of a dye cargo molecule. Formation and reaction of thiols resulted in *ca.* 80% release of umbelliferone in 4 h of sonication at 20 kHz with a considerably faster intramolecular activation than the previously described intermolecular activation system. Similar release trends were observed when the anti-cancer drug camptothecin was employed. The result of cell proliferation assays indicated that the cytotoxicity increased with sonication time due to increasing release. The important feature of this unified design was that non-toxic delivery platform POEGMEA shielded the conjugated drug and activation system within the random coil before sonication, causing a low activity and resistance to endogenous reduction of the disulfides in the non-activated form. This was a significant development allowing the applicability of the system in the presence of cells. However, the prolonged sonication conditions were not found to be compatible with mammalian HeLa cells.

In the next step of improving the intramolecular mechanochemical release system, the real-time tracking of drug activity was achieved by replacing one drug molecule with a fluorescent reporter on the mechanophore (Fig. 3c).^85^ Thereby, the reporter and the drug molecule were released simultaneously for spatiotemporal imaging of the delivery and uptake. The theranostic performance was investigated qualitatively with confocal laser scanning microscopy as well as quantitatively with fluorescence-activated cell sorting. Together with the cell proliferation assays and liquid chromatography-mass spectrometry data of released molecules, the theranostic functionality of the disulfide based mechanochemical activation was successfully verified.

Although many drugs carry hydroxyl groups, the scope of the available functional groups was limited to this moiety. This limitation was approached by investigating disulfide-centered systems with amino-naphthalimides to form β-carbamates as linkers instead of carbonates.^86^ The rates of force-induced intramolecular 5-*exo-trig* cyclization from β-carbamates were expectedly found to be considerably lower than the respective β-carbonate derivatives due to the poor leaving group properties of amines.

**Fig. 4:**
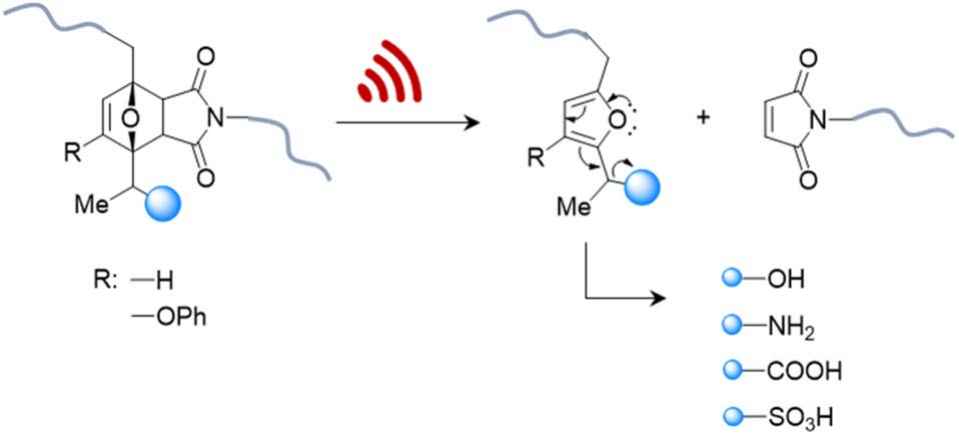
US-induced cargo release from 2-furylcarbinol mechanophore *via* retro Diels–Alder reaction followed by fragmentation cascade.^87^ The cargo scope includes alcohols (aryl/alkyl), amines (aryl/alkyl), carboxylic acids, and sulfonic acids.

Therefore, systems based on masked 2-furylcarbinol mechanophores developed by Robb and coworkers may be better suited since these allow the release of alcohols, phenols, alkylamines, arylamines, carboxylic acids, and sulfonic acids (Fig. 4).^87^ The ability to control the rate of release was demonstrated by fine-tuning the molecular structure of the furan–maleimide mechanophore with the guidance of density functional theory calculations.

This demonstrated that substituents on the 3-position of the 2-furylcarbinol significantly affected the reactivity of the molecular release through decomposition of the unstable furfuryl carbonate motif after mechanically initiated retro Diels–Alder reaction. Combined with the influence of an α-methyl substituent, the activation energy barrier of fragmentation and thermal stability could be modulated by varying the substituents without negatively affecting the mechanochemical performance.

**Fig. 5:**
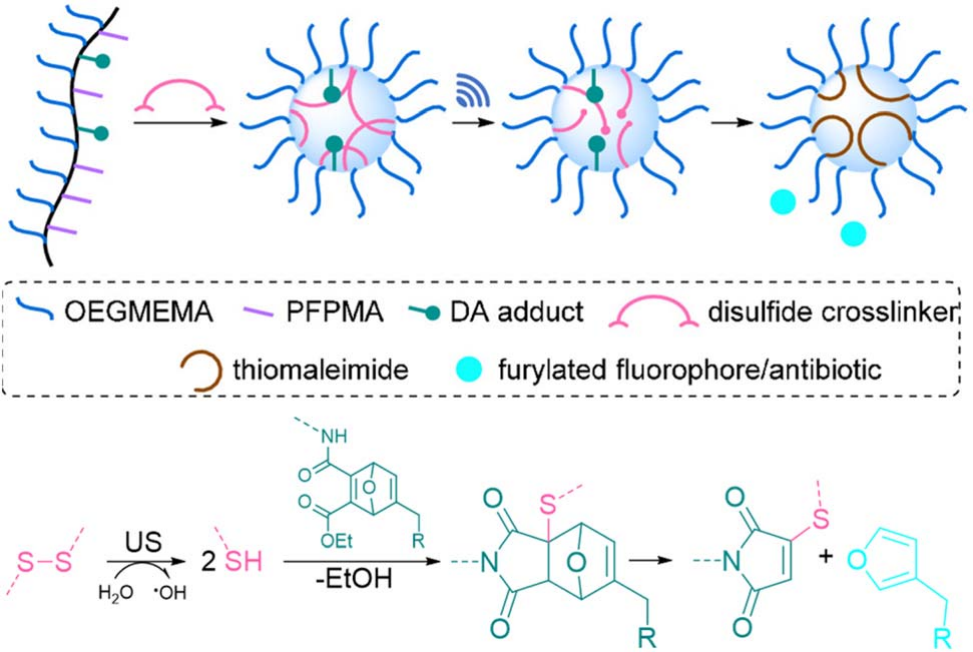
Schematic illustration of payload release from mechanoresponsive microgels. US cleaves disulfide crosslinkers resulting in a Michael addition to a DA adduct that releases a furan dansyl or trimethoprim. Adapted with permission from ref. 56. Copyright the authors.

All of the systems described above had the common drawback that activation requires long sonication times, up to multiple hours, which is not realistic for biomedical purposes. Herrmann, Göstl, and coworkers hypothesized that changing the topology of polymers could improve the activation efficiency also in the context of drug delivery and developed a microgel carrier^57^ to load and release drug molecules.^56^ The release mechanism was based on the scission of disulfide crosslinkers to yield thiols that undergo Michael-type addition to the DA adduct liberating the payload. Payload molecules were first attached on methacrylates through a Diels–Alder reaction of furylated molecules including drugs (Fig. 5). These methacrylates were used as monomers, combined with hydrophilic oligo(ethylene glycol)methyl ether methacrylate (OEGMEMA) and reactive pentafluorophenyl methacrylate (PFPMA) to yield linear copolymers. Consecutive crosslinking with cystamine disulfides of the PFPMA moieties formed microgels with sizes of hundreds of nm. After the releasing conditions were optimized by using microgels loaded with fluorescent furylated dansyl, antimicrobial activity was investigated with furylated antibiotic trimethoprim as loaded prodrug against Gram-positive and Gram-negative bacteria. The authors determined an US-induced released fraction reaching 42% in 5 min of sonication, followed by 48 h of incubation. Minimal inhibitory concentration (MIC) tests against *S. aureus* demonstrated that microgels were mostly inactive before sonication, because of the well encapsulated and shielded trimethoprim. After 5 min of US, MIC values decreased as low as pristine TMP, despite the fact that furylation decreased the potency of the antibiotic. Thereby, implementation of disulfide-based activation to microgels circumvented the need of long sonication times, which may be a key factor for future biomedical applications.

## Non-covalent mechanophore systems

### Nucleic acid-based host–guest systems

**Fig. 6:**
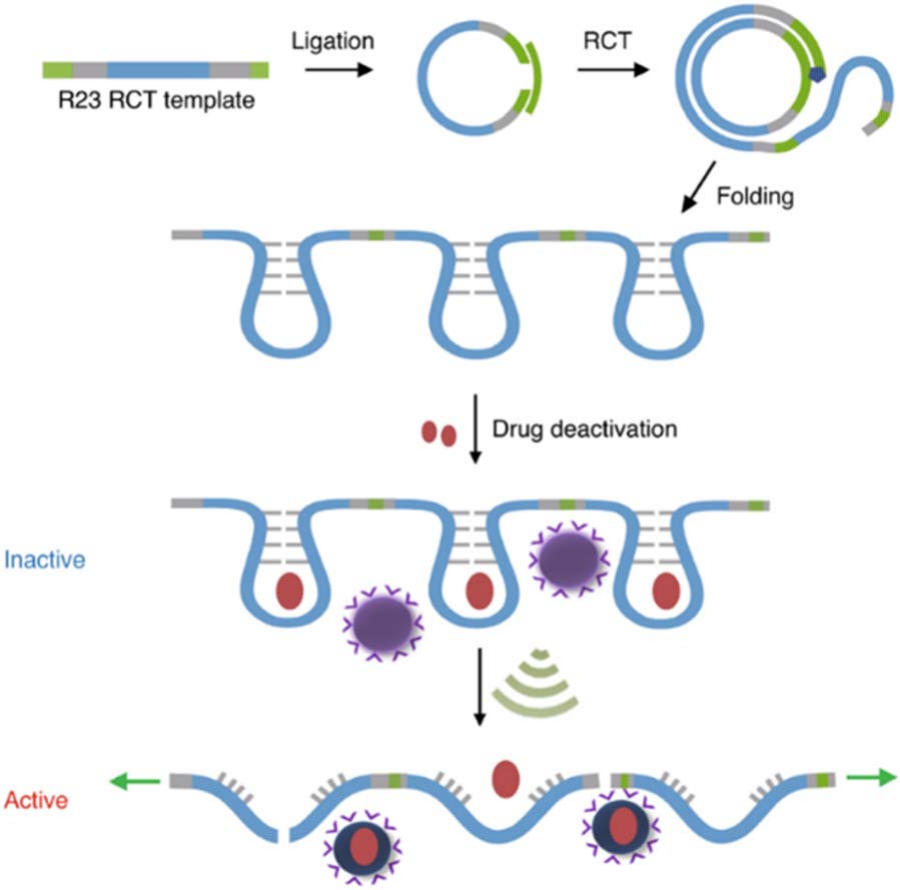
Preparation of R23 polyaptamers by RCT from circular template and antibiotic releasing mechanism based on US-induced stretching and scission. Reprinted with permission from ref. 61. Copyright the authors under exclusive license to Springer Nature Limited.

Covalent mechanophores tend to require high forces to achieve bond scission in turn requiring long exposure times, which is detrimental for use in a biomedical context. Therefore, non-covalent host–guest systems are rendered attractive mechanophores, since they may disassemble under lower forces. One approach to implement host–guest mechanophore systems relies on nucleic acid superstructures (aptamers, APTs) that bind their cargo strongly by specific non-covalent interactions. APTs are short and single-stranded segments of DNA or RNA and can bind to specific target molecules. As an example, R23 RNA APT was previously proven to bind aminoglycoside antibiotics, *e.g.*, neomycin B (NeoB) and paramomycin thus inhibiting their activity.^88^ This APT-antibiotic complex was used as the first example of an RNA-based mechanophore to enable mechanochemical activation.^61^ Since the mechanochemical activity highly depends on molar mass, repeating RNA strands were prepared by rolling circle transcription (RCT), yielding ultra-long polyaptamers (*P*_APT_) with hydrodynamic sizes of 0.1–1 μm (Fig. 6). *P*_APT_ was loaded with fluorescently labelled antibiotics and tested for its deactivation ability and US-induced (20 kHz) release. Solution experiments revealed that the NeoB@*P*_APT_ complex was as inert as NeoB@APT, however the former was able to release 80% of the NeoB cumulatively through mechanochemical scission of non-covalent host–guest interactions and covalent scission of the nucleic acid backbone, whereas the latter showed no antibiotic activity due to lacking mechanochemical activity. *In situ* experiments were also performed with *S. aureus* to compare MIC values after US-induced activation. 10 min of sonication of NeoB@*P*_APT_ resulted in a MIC of 8 μg mL^−1^ and further 20 min sonication allowed to reach the MIC of pristine NeoB. On the other hand, the control groups with NeoB@APT under 30 min sonication, NeoB@*P*_APT_ before sonication, and sonication alone showed no considerable effect on *S. aureus* viability. This system acted as the first proof of principle work for the US-induced activation mechanism for antimicrobial activity of antibiotics and moreover allowed to increase the loaded drug content due to a multi-mechanophore architecture along the polymer backbone. The high molar masses of the *P*_APT_ led to a pronounced and swift mechanochemical response compared to common linear synthetic polymers.

**Fig. 7:**
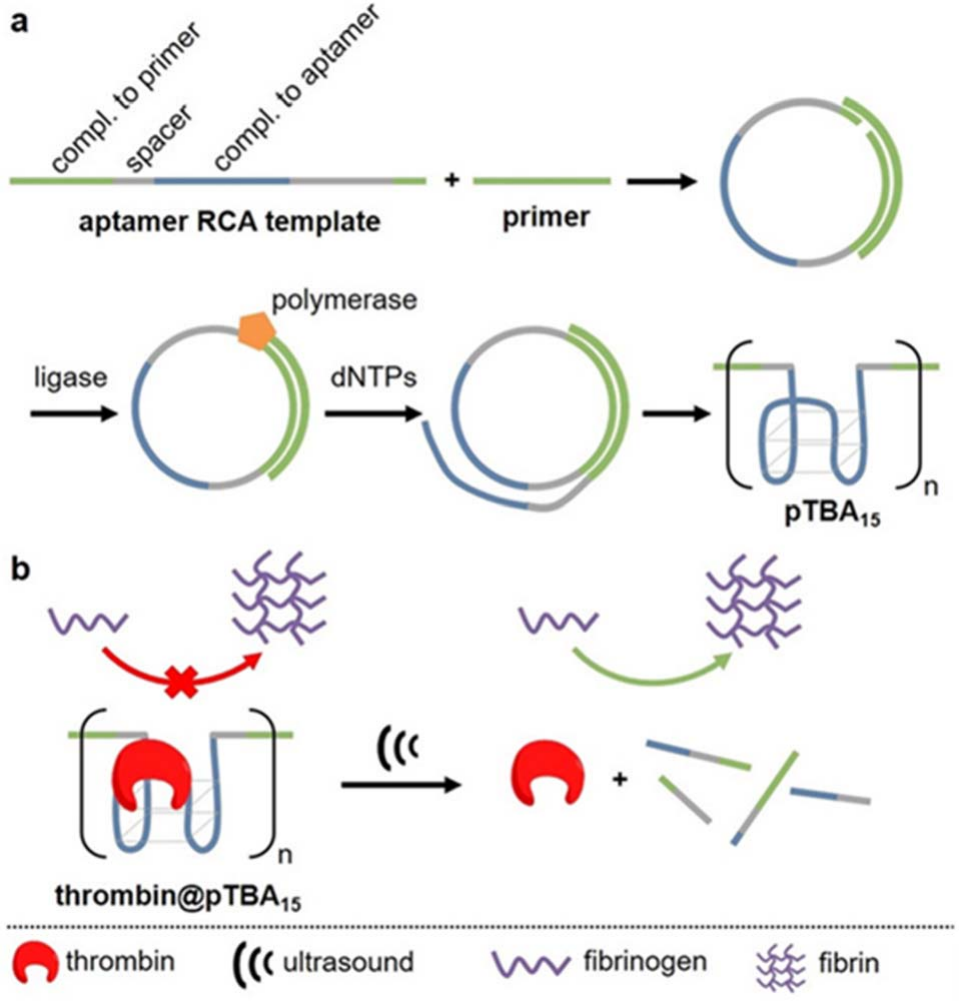
Schematic representation of US-induced activation of thrombin. (a) Synthesis of pTBA_15_ by RCA from circular template. (b) Release of thrombin that catalyzes fibrinogen formation from fibrin. Reproduced with permission from ref. 62. Copyright the authors.

APT protection of cargo was then also shown to be useful for the release of enzymes.^62^ Therefore, thrombin and its deactivating APT (TBA_15_) were used as enzyme-inhibitor couple. A high molar mass version of the APT (pTBA_15_) was prepared by rolling circle amplification so that the host unit would be responsive to US and could be loaded with thrombin (Fig. 7). The release and activation of thrombin were validated by catalytic conversion of fibrinogen into fibrin. It is important to note that polyaptamers were sonicated for 6 min with a low-intensity focused US (LIFU) setup at 5 MHz to yield approximately 80% activation. Hence, the authors successfully demonstrated an US activation design with a biomedically approvable setup of low US doses that could pave a way to improve current pharmaceutical control mechanisms. This activation was further adapted to an aptamer–NP system (*vide infra*).

**Fig. 8:**
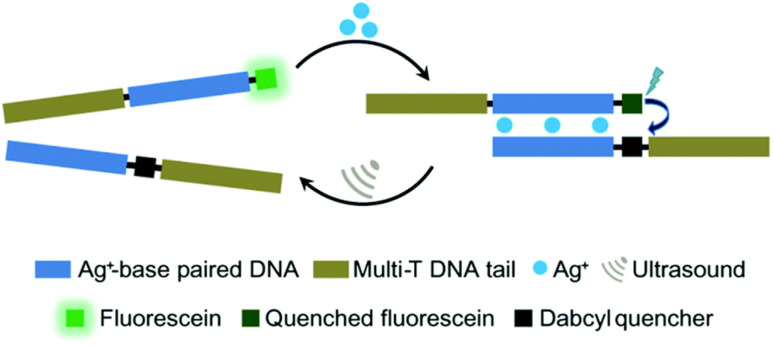
US-induced regulation of C–Ag^+^–C-base-pair interactions for DNA hybridization. The de-hybridization is reversible. Reprinted with permission from ref. 92. Copyright 2021 Royal Society of Chemistry.

Moreover, oligonucleotides that allow the construction of host–guest complexes with cations, such as Ag^I^, Hg^II^, Au^III^, and Cu^II^, for functional and structural applications^89–91^ were investigated as mechanophores (Fig. 8). Recently, Herrmann and coworkers used US to regulate such metallo-base-pair interactions selectively and reversibly.^92^ The system was based on well-established C–Ag^+^–C-base-pairs with long multi-T DNA domains attached to allow US-activation. Moreover, the strands were labelled with fluorescein and its quencher to monitor the hybridization–dissociation process. First, hybridization was monitored by fluorescence quenching as the concentration of Ag^+^ increased in the medium. The US-induced dissociation was observed by fluorescence recovery. The tail length had a significant influence on the association–dissociation mechanism. The shorter the multi-T tail, the stronger the Ag^+^ mediated hybridization, however short tail (0 and 10T) pairs did not dissociate under 70 min US (20 kHz). Fluorescence recovery, signaling the dissociation, was observed with 30T and 50T tail pairs and up to 80% recovery was reported.

### Supramolecular assemblies

In addition to the polynucleic acid-based systems presented above, other supramolecular constructs have proven useful for the force-induced release or activation of drugs that are non-covalently anchored within their deactivating macromolecular complex. The supramolecular bond is individually weaker than a covalent bond and intrinsically features reversible and dynamic character. Yet, supramolecular bonds, and specifically the synergy of multiple supramolecular bonds, can be strong enough to bind complex assemblies by, *e.g.*, hydrogen bonds, metal–ligand coordination, hydrophobic–hydrophilic interactions, and others. Similar to the metallo-base-pair-interactions (*vide supra*), metal–ligand coordination can be used as crosslinking strategy for polymer networks and reversible cation exchange was reported under mechanical force.^93^ Recently, the H-bond-based supramolecular dimer of the antibiotic vancomycin (Van) and its complementary peptide target sequence, Cys-Lys-Lys(Ac)-d-Ala-d-Ala (DADA) was identified as force-responsive motif by Herrmann, Göstl, and coworkers. The molecular pair was used to activate antimicrobial activity by ultrasound anchored in different architectures including polymer–NP (PN),^61^ NP–NP (NN),^61^ and polymer brush (PB)^51^ systems as a means to overcome antibiotic resistance by only activating antibiotics locally.

**Fig. 9:**
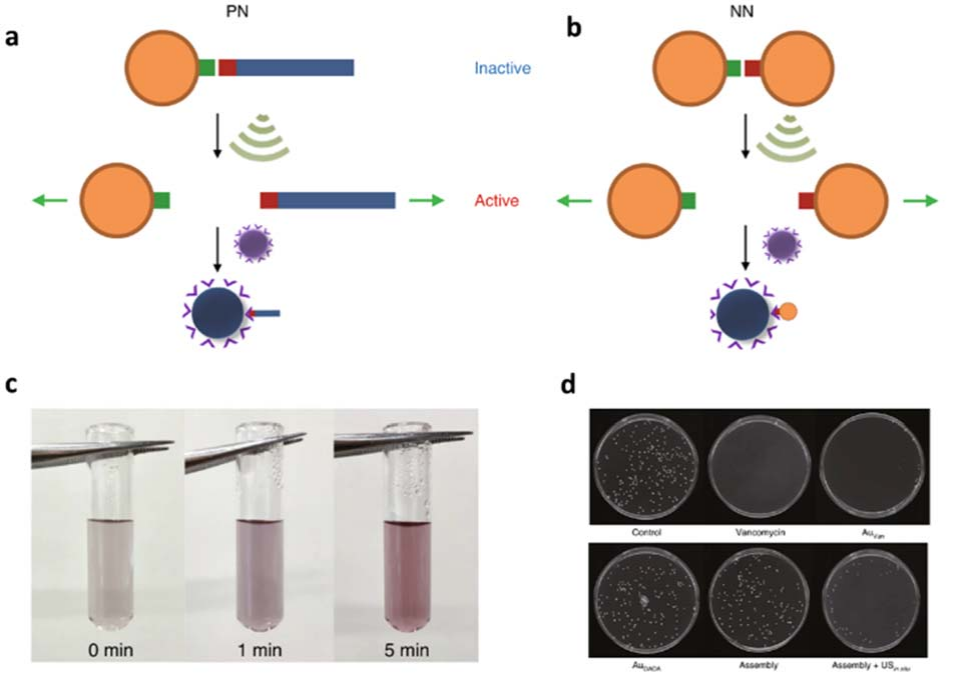
Schematic illustration of US-induced antimicrobial activity based on Van–DADA (red-green) assemblies in (a) polymer–nanoparticle PN and (b) nanoparticle–nanoparticle NN systems. (c) Color change in solution of NN before and after sonication (0, 1, and 5 min). (d) Agar Petri dishes demonstrating the US-induced antibiotic activity: *S. aureus* treated with PBS, pristine Van, Au–Van, Au–DADA, NN before sonication and NN after *in situ* sonication, respectively. Adapted with permission from ref. 61. Copyright the authors under exclusive license to Springer Nature Limited.

Utilization of AuNPs as transmitter of the shear force is a promising approach for medical applications because they are relatively stable, biocompatible, easy to synthesize or functionalize, besides possessing tunable optical, electronic, and physicochemical characteristics that render them unique among not only NPs but also other delivery modalities.^94^ For the preparation of PN assemblies, AuNPs were modified with DADA and Van was extended with POEGMEMA through Cu-mediated controlled radical polymerization. Finally, both were incubated together to yield PN nanostructures that were tested on *S. aureus* (Fig. 9a).

*In situ* sonication experiments showed that PN assemblies successfully released polymer chains (10 and 20 kDa) carrying Van into solution and thereby the MIC values were lowered significantly. Concomitantly, control experiments with DADA–AuNPs under US, small molecular Van–DADA–AuNPs under US, and polymer Van–DADA–AuNPs before US application did not exhibit any antimicrobial activity. Similarly, NN assemblies were prepared by replacing Van-terminated POEGMEMA with relatively smaller Van-decorated AuNPs (Fig. 9b). A comparable trend for the US-induced antimicrobial activity was observed with a decrease of the MIC values with progressing sonication time. In contrast to Van-terminated polymer chains, Van-conjugated AuNPs showed MIC values almost identical to those of pristine Van. Thereby, a problem often encountered in polymer mechanochemistry was circumvented where the activated functional moiety is terminally dangling on the mechanochemically generated chain fragment thus being screened within the hydrodynamic coil of the polymer and therefore exhibiting lower activities. In addition, the disassembly process was monitored by the change in color as AuNPs' plasmonic absorbance is altered with aggregate size and shape.

**Fig. 10:**
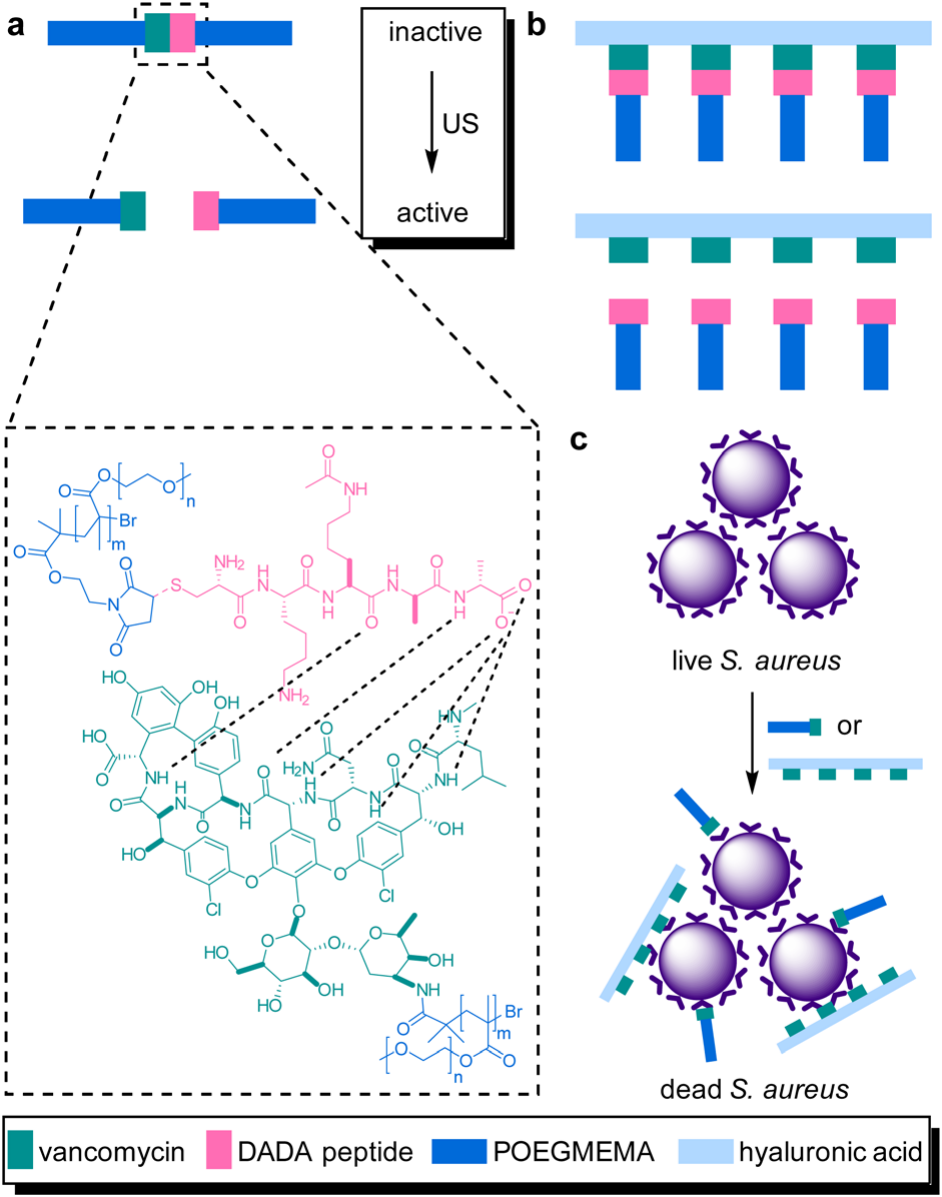
Van–DADA adduct based prodrug embedded in (a) the central site of linear polymer of POEGMEMA, (b) HA polymer brush. US induces the activation by breaking the weak H-bonding between DADA peptide and Van, (c) releasing the Van–HA that binds on the *S. aureus* and inhibits cell wall synthesis. Reprinted with permission from ref. 51. Copyright 2021 the authors.

The systematic problems encountered in the pioneering work in the field of sonopharmacology, such as low drug content and low mechanochemical efficiency, were then addressed by incorporating the Van–DADA couple into polymer brushes. Polymer brushes were prepared with Van-functionalized hyaluronic acid (HA) as backbone and DADA-terminated POEGMEMA chains as brushes in a “grafting to” approach (Fig. 10). Thereby, screening of Van by the hydrodynamic coil of the polymer chain was inhibited while multivalency effects of Van were enabled both increasing the drug potency. Moreover, MIC tests revealed that only 5 min of sonication (20 kHz) disassembled the supramolecular brush to activate the antimicrobial behavior as efficient as pristine Van. In contrast, the MIC value of the intact brushes was found to be one order of magnitude higher. Thereby the mechanochemical efficiency of the system was improved while simultaneously increasing the potency and loaded content of the activated drug construct.

In the previous section, the release from deactivating polyaptamer complexes by US was discussed. This was expanded to aptamer–NP-based supramolecular assemblies using LIFU at 5 MHz.^62^ Therefore, AuNPs were functionalized with thiolated split aptamers (TBA_15_), inducing an aggregation of nanoparticles in the presence of thrombin to inhibit its activity. The aggregated NPs underwent disassembly under US and activation performance of enzyme was comparable to those of the polyaptamer system. However, in stark contrast to the latter system, the disassembly process was found to be reversible and could be cycled without substantial fatigue.

**Fig. 11:**
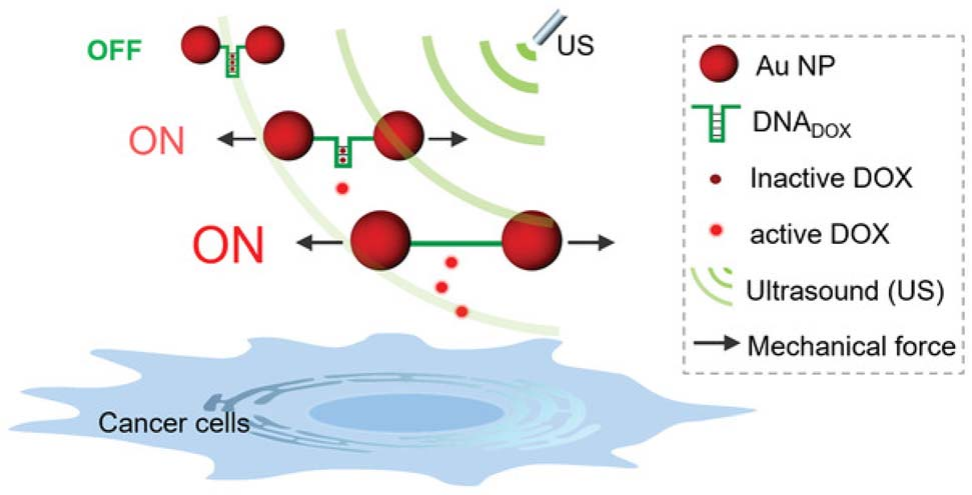
Representation of the Au–DNA_DOX_ complex releasing DOX under US irradiation for controlled cancer cell inhibition. AuNPs transmit the shear force to stretch the bridge and dissociate the DNA_DOX_ dimer. Reprinted from ref. 95. Copyright 2022 the authors.

Following their pivotal reports on AuNPs as transmitter of the shear force, Huo, Herrmann, and coworkers constructed another mechanoresponsive motive to further extend the scope of the US-induced release mechanisms.^95^ Two citrate-protected AuNPs were connected by a specific single-stranded (ss) DNA sequence that can hybridize into a hairpin structure to form double-stranded (ds) DNA (Fig. 11). The DNA sequence contained 5′-GC-3′ repetitions to which DOX can non-covalently intercalate, resulting in deactivation and fluorescence quenching. The yield of the drug loading efficiency was determined as 64% based on fluorescence quenching of the drug, considering ∼10 loading sites available per ssDNA. Upon US irradiation, the dissociation of the base pair interactions in the Au–DNA dimer structure was observed with the help of transmission electron microscopy. The drug release was also verified by fluorescence recovery of the DOX, indicating a yield of 60% in 30 min sonication. Moreover, addition of a complementary ssDNA into the sonication medium increased the yield of release up to 70%, because of the competitive hybridization that prevents recombination. MTT assays were further performed to investigate the inhibition of cancer cell proliferation, demonstrating primarily a decrease in drug activity of Au–DNA_DOX_ compared to pristine DOX due to intercalation of DOX within the Au–DNA dimer structure, and thereafter a decrease in cell viability when LNCaP cells treated with *ex situ* sonicated samples due to successful mechanochemical activation. This US-responsive Au–DNA nanoswitch provided a blueprint for construction of facile structured NP systems that can be generalized to other drugs by tuning DNA sequences.

**Fig. 12:**
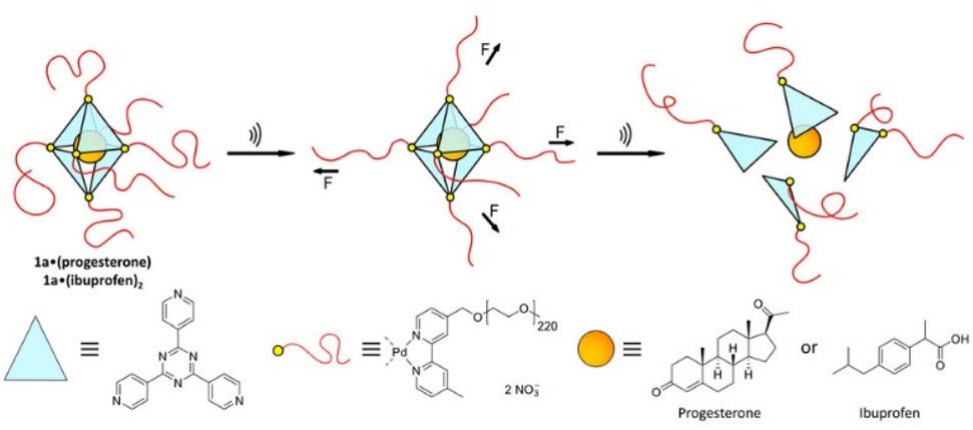
Schematic illustration of US-induced fragmentation of and subsequent release from the PEG-functionalized octahedral Pd^II^_6_(TPT)_4_ cage encapsulating drugs non-covalently. Reprinted from ref. 96. Copyright the authors.

Universal applicability of carrier systems is a general issue of drug delivery. Schmidt, Göstl, and coworkers devised a supramolecular, force-responsive cargo system that encapsulates virtually any hydrophobic drug (Fig. 12).^96^ The drugs were non-covalently caged in their pristine form without additional functionalization for assembly. The supramolecular cage was prepared by coordination of Pd–bipyridine complex with 2,4,6-tri(pyridin-4-yl)-1,3,5-triazine (TPT) to yield Pd_6_(TPT)_4_. The bipyridine ligands carried PEG chains to enable US-induced fragmentation of the cage. The resulting octahedral complex with hydrophobic cage was loaded with progesterone or ibuprofen in aqueous media and irradiated with US (20 kHz, 1 h) in H_2_O. ^1^H-NMR experiments indicated the fragmentation-induced release of ibuprofen, even though the exact fragmentation mechanism remained unclear. Yet, unmodified Pd_6_(TPT)_4_ did not release any ibuprofen under similar sonication conditions, revealing the mechanochemical origin of the release mechanism. Successful release of progesterone under identical conditions supported the universality of the carrier system.

## Conclusions

US is an external stimulus that enables control over drug activation and release as well as the regulation of biochemical processes thereby offering promising potential for future biomedical and clinical applications. Alongside other controlled delivery and release systems, this is particularly crucial for drugs that spawn resistances over time, such as antibiotics, or highly toxic drugs, such as anti-cancer medication, that result in severe off-target side effects. While US has been employed to enhance drug delivery exploiting its thermal and non-directed mechanical effects, recent developments show that US can even be used as a molecular scalpel to cleave selectively specific bonds for the activation or the release of drug molecules in biological systems. Here, we highlighted these concepts and developments that are based on the principles of polymer mechanochemistry. Hence, here we would like to define the term sonopharmacology. Sonopharmacology describes the spatial and temporal application of ultrasound for switching “on” or “off” an active pharmaceutical ingredient by the cleavage or rearrangement of specific covalent or non-covalent bonds, namely mechanophores that are embedded in polymers and macromolecules, using the principles of polymer mechanochemistry.

The first examples shaping the framework of drug activation and release systems are linear polymers with mechanophores embedded in the center position, where bond scission and subsequent cascade reactions release the desired drugs. These simple yet innovative systems provide the unique opportunity to gain spatiotemporal control with molecular precision over the release and activation processes. This is complemented by ongoing efforts to extend the cargo scope towards pristine or functionalized payload molecules towards generalized approaches and adaptable release kinetics.

However, fundamental obstacles must be overcome before biomedical or even clinical applications of such systems move within reach. Systems based on linear polymer chains suffer from low drug loading content since the whole macromolecular framework serves as a scaffold to transduce force to a single central mechanophore. Complicating this disadvantage, the mechanochemical efficiency is inversely proportional to the molar mass and contour length of the polymer chain. This means that a faster and thus biologically more benign release further reduces the mass fraction of incorporated payload. The first circumventions for this problem rely on multi-mechanophore architectures that can be loaded with significantly more payload molecules. Alongside, the influence of polymer architecture and topology is exploited using super-responsive polymer structures, such as polymer brushes or colloidal hydrogel networks as carriers. Thereby, sonication durations are drastically reduced minimizing the necessary energy input further paving the way towards better compatibility with biological systems.

Another currently limiting factor in sonopharmacology is the employed US sources. Traditional sonication conditions in polymer mechanochemistry use low frequency US in the kHz range that leads to inertial cavitation which, in turn, induces cell death. Therefore, first advances using clinically established ultrasound sources, such as HIFU or LIFU, with MHz frequencies for the activation of polymer mechanochemical transformations are being explored in conjunction with super-responsive polymer architectures and enzymes. Thereby, crucial steps towards the application of the sonopharmacology method in a biomedical context are being taken. We thus anticipate that these advancements will be further developed to biomedical demands eventually enabling their application *in vivo*. Since US is an established modality in the clinic, we believe that future research will establish sonopharmacology as a powerful therapeutic and theranostic tool in the realm of medicine.

## Author contributions

R. G. and A. H. conceived the topic of the review and supervised the work. D. Y. designed the structure of the review and wrote the manuscript. R. G. contributed to the editing and reviewing of this work. All the authors participated in the discussion and revision of the manuscript.

## Conflicts of interest

There are no conflicts to declare.

## Supplementary Material

## References

[cit1] Longley D., Johnston P. (2005). J. Pathol..

[cit2] Carlet J., Collignon P., Goldmann D., Goossens H., Gyssens I. C., Harbarth S., Jarlier V., Levy S. B., N'Doye B., Pittet D., Richtmann R., Seto W. H., van der Meer J. W., Voss A. (2011). Lancet.

[cit3] Gunnarsson L., Snape J. R., Verbruggen B., Owen S. F., Kristiansson E., Margiotta-Casaluci L., Österlund T., Hutchinson K., Leverett D., Marks B., Tyler C. R. (2019). Environ. Int..

[cit4] Melo S. R. d. O., Homem-de-Mello M., Silveira D., Simeoni L. A. (2014). J. Pharm. Sci. Technol..

[cit5] Edwards I. R., Aronson J. K. (2000). Lancet.

[cit6] Tibbitt M. W., Dahlman J. E., Langer R. (2016). J. Am. Chem. Soc..

[cit7] Esser-Kahn A. P., Odom S. A., Sottos N. R., White S. R., Moore J. S. (2011). Macromolecules.

[cit8] Mi P. (2020). Theranostics.

[cit9] Choi S.-W., Zhang Y., Xia Y. (2010). Angew. Chem., Int. Ed..

[cit10] Velema W. A., Szymanski W., Feringa B. L. (2014). J. Am. Chem. Soc..

[cit11] Hüll K., Morstein J., Trauner D. (2018). Chem. Rev..

[cit12] Cardoso V. F., Francesko A., Ribeiro C., Bañobre-López M., Martins P., Lanceros-Mendez S. (2018). Adv. Healthcare Mater..

[cit13] Mitragotri S. (2005). Nat. Rev. Drug Discovery.

[cit14] Lentacker I., Smedt S. C. D., Sanders N. N. (2009). Soft Matter.

[cit15] Wells P. N. T. (2006). Phys. Med. Biol..

[cit16] Su C., Ren X., Nie F., Li T., Lv W., Li H., Zhang Y. (2021). RSC Adv..

[cit17] Yang C., Li Y., Du M., Chen Z. (2019). J. Drug Targeting.

[cit18] Cai X., Jiang Y., Lin M., Zhang J., Guo H., Yang F., Leung W., Xu C. (2020). Front. Pharmacol..

[cit19] Lattwein K. R., Shekhar H., Kouijzer J. J. P., van Wamel W. J. B., Holland C. K., Kooiman K. (2020). Ultrasound Med. Biol..

[cit20] Brosh D., Miller H. I., Herz I., Laniado S., Rosenschein U. (1998). Int. J. Cardiovasc. Interv..

[cit21] Kiessling F., Fokong S., Bzyl J., Lederle W., Palmowski M., Lammers T. (2014). Adv. Drug Delivery Rev..

[cit22] Phenix C. P., Togtema M., Pichardo S., Zehbe I., Curiel L. (2014). J. Pharm. Pharm. Sci..

[cit23] Geers B., Dewitte H., De Smedt S. C., Lentacker I. (2012). J. Controlled Release.

[cit24] de Matos M. B. C., Deckers R., van Elburg B., Lajoinie G., de Miranda B. S., Versluis M., Schiffelers R., Kok R. J. (2019). Front. Pharmacol..

[cit25] Kopechek J. A., Haworth K. J., Radhakrishnan K., Huang S.-L., Klegerman M. E., McPherson D. D., Holland C. K. (2013). Ultrason. Sonochem..

[cit26] Yang P., Li D., Jin S., Ding J., Guo J., Shi W., Wang C. (2014). Biomaterials.

[cit27] Rapoport N. (2012). WIREs Nanomed. Nanobiotechnol..

[cit28] Helfield B., Zou Y., Matsuura N. (2021). Front. Phys..

[cit29] Marin A., Muniruzzaman M., Rapoport N. (2001). J. Controlled Release.

[cit30] Ahmed S. E., Martins A. M., Husseini G. A. (2015). J. Drug Targeting.

[cit31] Chandan R., Mehta S., Banerjee R. (2020). ACS Biomater. Sci. Eng..

[cit32] Kiessling F., Fokong S., Koczera P., Lederle W., Lammers T. (2012). J. Nucl. Med..

[cit33] Kooiman K., Vos H. J., Versluis M., de Jong N. (2014). Adv. Drug Delivery Rev..

[cit34] Lentacker I., De Cock I., Deckers R., De Smedt S. C., Moonen C. T. W. (2014). Adv. Drug Delivery Rev..

[cit35] Keller S. B., Averkiou M. A. (2022). Bioconjugate Chem..

[cit36] Wu P., Jia Y., Qu F., Sun Y., Wang P., Zhang K., Xu C., Liu Q., Wang X. (2017). ACS Appl. Mater. Interfaces.

[cit37] Chowdhury S. M., Abou-Elkacem L., Lee T., Dahl J., Lutz A. M. (2020). J. Controlled Release.

[cit38] O'Neill R. T., Boulatov R. (2021). Nat. Rev. Chem..

[cit39] Chen Y., Mellot G., van Luijk D., Creton C., Sijbesma R. P. (2021). Chem. Soc. Rev..

[cit40] Traeger H., Kiebala D. J., Weder C., Schrettl S. (2021). Macromol. Rapid Commun..

[cit41] He S., Stratigaki M., Centeno S. P., Dreuw A., Göstl R. (2021). Chem.–Eur. J..

[cit42] Groote R., Jakobs R. T. M., Sijbesma R. P. (2013). Polym. Chem..

[cit43] Versaw B. A., Zeng T., Hu X., Robb M. J. (2021). J. Am. Chem. Soc..

[cit44] Küng R., Göstl R., Schmidt B. M. (2022). Chem.–Eur. J..

[cit45] Dougherty T. J., Gomer C. J., Henderson B. W., Jori G., Kessel D., Korbelik M., Moan J., Peng Q. (1998). JNCI, J. Natl. Cancer Inst..

[cit46] Nyborg W. L. (2001). Ultrasound Med. Biol..

[cit47] Stratigaki M., Göstl R. (2020). ChemPlusChem.

[cit48] May P. A., Moore J. S. (2013). Chem. Soc. Rev..

[cit49] May P. A., Munaretto N. F., Hamoy M. B., Robb M. J., Moore J. S. (2016). ACS Macro Lett..

[cit50] Schaefer M., Icli B., Weder C., Lattuada M., Kilbinger A. F. M., Simon Y. C. (2016). Macromolecules.

[cit51] Zou M., Zhao P., Huo S., Göstl R., Herrmann A. (2022). ACS Macro Lett..

[cit52] Peterson G. I., Noh J., Bang K.-T., Ma H., Kim K. T., Choi T.-L. (2020). Macromolecules.

[cit53] Sheiko S. S., Sun F. C., Randall A., Shirvanyants D., Rubinstein M., Lee H., Matyjaszewski K. (2006). Nature.

[cit54] Izak-Nau E., Demco D. E., Braun S., Baumann C., Pich A., Göstl R. (2020). ACS Appl. Polym. Mater..

[cit55] Izak-Nau E., Braun S., Pich A., Göstl R. (2022). Adv. Sci..

[cit56] Zou M., Zhao P., Fan J., Göstl R., Herrmann A. (2022). J. Polym. Sci..

[cit57] Schulte M. F., Izak-Nau E., Braun S., Pich A., Richtering W., Göstl R. (2022). Chem. Soc. Rev..

[cit58] Noh J., Peterson G. I., Choi T.-L. (2021). Angew. Chem., Int. Ed..

[cit59] Watabe T., Ishizuki K., Aoki D., Otsuka H. (2019). Chem. Commun..

[cit60] Watabe T., Aoki D., Otsuka H. (2021). Macromolecules.

[cit61] Huo S., Zhao P., Shi Z., Zou M., Yang X., Warszawik E., Loznik M., Göstl R., Herrmann A. (2021). Nat. Chem..

[cit62] Zhao P., Huo S., Fan J., Chen J., Kiessling F., Boersma A. J., Göstl R., Herrmann A. (2021). Angew. Chem., Int. Ed..

[cit63] Piermattei A., Karthikeyan S., Sijbesma R. P. (2009). Nat. Chem..

[cit64] Michael P., Binder W. H. (2015). Angew. Chem., Int. Ed..

[cit65] Campagna D., Göstl R. (2022). Angew. Chem., Int.
Ed..

[cit66] Löwe C., Weder C. (2002). Adv. Mater..

[cit67] Davis D. A., Hamilton A., Yang J., Cremar L. D., Van Gough D., Potisek S. L., Ong M. T., Braun P. V., Martínez T. J., White S. R., Moore J. S., Sottos N. R. (2009). Nature.

[cit68] Chen Y., Spiering A. J. H., Karthikeyan S., Peters G. W. M., Meijer E. W., Sijbesma R. P. (2012). Nat. Chem..

[cit69] Willis-Fox N., Rognin E., Aljohani T. A., Daly R. (2018). Chem.

[cit70] Ghanem M. A., Basu A., Behrou R., Boechler N., Boydston A. J., Craig S. L., Lin Y., Lynde B. E., Nelson A., Shen H., Storti D. W. (2021). Nat. Rev. Mater..

[cit71] Turksoy A., Yildiz D., Aydonat S., Beduk T., Canyurt M., Baytekin B., Akkaya E. U. (2020). RSC Adv..

[cit72] Pibiri I., Buscemi S., Palumbo Piccionello A., Pace A. (2018). ChemPhotoChem.

[cit73] Nonell S. and Flors C., Singlet Oxygen, The Royal Society of Chemistry, 2016, vol. 1

[cit74] Kim G., Wu Q., Chu J. L., Smith E. J., Oelze M. L., Moore J. S., Li K. C. (2022). Proc. Natl. Acad. Sci. U. S. A..

[cit75] Motterlini R., Otterbein L. E. (2010). Nat. Rev. Drug Discovery.

[cit76] Katsnelson A. (2019). ACS Cent. Sci..

[cit77] Otterbein L. E., Bach F. H., Alam J., Soares M., Tao Lu H., Wysk M., Davis R. J., Flavell R. A., Choi A. M. K. (2000). Nat. Med..

[cit78] Sun Y., Neary W. J., Burke Z. P., Qian H., Zhu L., Moore J. S. (2022). J. Am. Chem. Soc..

[cit79] Shi Z., Wu J., Song Q., Göstl R., Herrmann A. (2020). J. Am. Chem. Soc..

[cit80] Li Y., Nese A., Matyjaszewski K., Sheiko S. S. (2013). Macromolecules.

[cit81] Fritze U. F., von Delius M. (2016). Chem. Commun..

[cit82] Wang F., Burck M., Diesendruck C. E. (2017). ACS Macro Lett..

[cit83] Fritze U. F., Craig S. L., von Delius M. (2018). J. Polym. Sci., Part A: Polym. Chem..

[cit84] Cohen N. (1996). J. Phys. Chem. Ref. Data.

[cit85] Shi Z., Song Q., Göstl R., Herrmann A. (2021). Chem. Sci..

[cit86] Shi Z., Song Q., Göstl R., Herrmann A. (2021). CCS Chem..

[cit87] Hu X., Zeng T., Husic C. C., Robb M. J. (2021). ACS Cent. Sci..

[cit88] Bastian A. A., Marcozzi A., Herrmann A. (2012). Nat. Chem..

[cit89] Tanaka Y., Kondo J., Sychrovský V., Šebera J., Dairaku T., Saneyoshi H., Urata H., Torigoe H., Ono A. (2015). Chem. Commun..

[cit90] Heuberger B. D., Shin D., Switzer C. (2008). Org. Lett..

[cit91] Yamaguchi H., Šebera J., Kondo J., Oda S., Komuro T., Kawamura T., Dairaku T., Kondo Y., Okamoto I., Ono A., Burda J. V., Kojima C., Sychrovský V., Tanaka Y. (2014). Nucleic Acids Res..

[cit92] Huo S., Zhou Y., Liao Z., Zhao P., Zou M., Göstl R., Herrmann A. (2021). Chem. Commun..

[cit93] Balkenende D. W. R., Coulibaly S., Balog S., Simon Y. C., Fiore G. L., Weder C. (2014). J. Am. Chem. Soc..

[cit94] Hu X., Zhang Y., Ding T., Liu J., Zhao H. (2020). Front. bioeng. biotechnol..

[cit95] Huo S., Liao Z., Zhao P., Zhou Y., Göstl R., Herrmann A. (2022). Adv. Sci..

[cit96] Küng R., Pausch T., Rasch D., Göstl R., Schmidt B. M. (2021). Angew. Chem., Int. Ed..

